# Leveraging Sub-national Collaboration and Influence for Improving Animal Health Surveillance and Response: A Stakeholder Mapping in Tanzania

**DOI:** 10.3389/fvets.2021.738888

**Published:** 2021-12-13

**Authors:** Janeth George, Barbara Häsler, Erick V. G. Komba, Calvin Sindato, Mark Rweyemamu, Sharadhuli I. Kimera, James E. D. Mlangwa

**Affiliations:** ^1^Department of Veterinary Medicine and Public Health, Sokoine University of Agriculture, Morogoro, Tanzania; ^2^SACIDS Foundation for One Health, Sokoine University of Agriculture, Morogoro, Tanzania; ^3^Department of Pathobiology and Population Sciences, Veterinary Epidemiology, Economics, and Public Health Group, Royal Veterinary College, Hatfield, United Kingdom; ^4^National Institute for Medical Research, Tabora Research Centre, Tabora, Tanzania

**Keywords:** stakeholder mapping, stakeholder analysis, collaboration, animal health surveillance, Tanzania, stakeholder influence

## Abstract

Animal health surveillance plays a vital role in ensuring public health, animal welfare, and sustainable food production by monitoring disease trends, early detecting (new) hazards, facilitating disease control and infection, and providing data for risk analysis. Good stakeholder collaboration across the sector can lead to better communication, better science and decision-making and more effective surveillance and response. An understanding of relevant stakeholders, their interests and their power can facilitate such collaboration. While information on key stakeholders in animal health surveillance is available at the national level in Tanzania, it is missing at the subnational level. The study aimed to explore the existing stakeholders' collaborations and influences at the subnational level through stakeholder mapping and to determine potential leverage points for improving the national animal health surveillance system. A qualitative design was used, involving consultative workshops with government animal health practitioners in Sumbawanga, Sikonge and Kilombero districts of Tanzania from December 2020 to January 2021. Data were collected using an adapted USAID stakeholder collaboration mapping tool with the following steps: (i) Define the objective (ii) Identify all stakeholders (iii) Take stock of the current relationships (iv) Determine resource-based influence (v) Determine non-resource based influence and (vi) Review and revise the collaboration map. Forty-five stakeholders were identified in all three districts and grouped into four categories: private sector and non-government organizations (*n* = 16), government (*n* = 16), community (*n* = 9) and political leaders (*n* = 4). Animal health practitioners had a stronger relationship with community stakeholders as compared to other categories. The results also showed that most of the stakeholders have non-resource-based influence compared to resource-based influence. The private sector and non-government organizations have a relatively higher number of resource-based influential stakeholders, while political leaders have more non-resource-based influence. The mapping exercise demonstrated that the system could benefit from community mobilization and sensitization, resource mobilization and expanding the horizon of surveillance data sources. Some of the leverage points include integration of surveillance activities into animal health services, clear operational processes, constant engagement, coordination and incentivization of stakeholders. The diversity in the identified stakeholders across the districts suggests that collaborations are contextual and socially constructed.

## Introduction

Animal health surveillance plays a vital role in ensuring public health, animal welfare, and sustainable food production by monitoring disease trends, detecting (new) hazards and unusual events, facilitating disease control and infection, and providing data for risk analysis (1). A functional and efficient surveillance system requires collaborative efforts from relevant stakeholders working toward a common goal with a sense of shared responsibility. The stakeholders include public and private veterinary service providers, farmers and other private and non-government stakeholders operating on the ground (2). Early detection and reporting of animal disease events fundamentally depend on people primarily working with animals throughout the production system (3), especially in the countries where the systems are not very mechanized, and observation of animals is crucial. Stakeholders are defined as any group or individuals who are affected by or could affect a particular course of action or decision (4). In health surveillance, these are the persons or organizations that contribute to, use and benefit from surveillance (5). Stakeholders can further be categorized into primary and secondary stakeholders (6). A primary stakeholder in animal health surveillance is a person or group essential for surveillance operations, including veterinary practitioners, livestock farmers, commercial farms, field officers, community representatives, government authorities, regulatory bodies, meat inspectors and veterinary laboratories. A secondary stakeholder in animal health surveillance is a person or group who is not essential in the operations of surveillance but may be interested in it, such as veterinary pharmaceuticals, research institutions, politicians and non-government organizations.

Stakeholder collaboration is defined as a mutually beneficial relationship between two or more parties who work toward common goals by sharing responsibility, authority, and accountability for achieving results (7). Collaboration helps to identify shared areas of interest and potential cooperation and avoid duplication of efforts (8). Several studies have demonstrated the importance of multiple stakeholders involved in disease control (9, 10) and successful surveillance programmes (11, 12). The increased need for rapid disease identification and prevention of epidemics has steered stakeholders' interest for better communication, better science and decision-making (12). However, motivation of stakeholders to participate in surveillance is influenced by the amount of information they have and the level of concern on the particular hazard (13). The interrelationship between actors involved in the animal health surveillance system can enhance or constrain the system; for example, acceptability of the system, reporting and information use for disease control (14). Thus, for the stakeholder collaboration to be successful, it is essential to understand their perception of surveillance benefits, the value of the collected epidemiological data, knowledge, motivation and trust (11).

An in-depth understanding of stakeholders can be achieved through thorough stakeholder mapping and analysis. Stakeholder analysis is an approach used to understand the system and its changes by identifying stakeholders and assessing their interest and influence within the system (12). Stakeholder analyses are now more critical than ever before because of increasingly interconnected nature of the world (15). Stakeholder mapping is a stakeholder analysis tool that involves creating pictures to clarify an organization's stakeholders (6). The tool has been used widely in project management (16, 17), policy (18, 19), education and natural resource management (20). In animal health, stakeholder mapping has been applied to analyze stakeholders in animal health surveillance systems (11, 12) and biosecurity governance (21). Stakeholder mapping and analysis help concerned parties to identify strengths, weaknesses and opportunities for future engagement based the relationship, interest and influence (22). It allows a better assessment of stakes of various interested parties, which may guide the design and governance of the system (23). There are several stakeholder mapping techniques, but the most common are the ones that plots stakeholders on the two-dimensional matrix/grid with two attributes: importance and influence or power and interest as its axes (15).

The government of Tanzania through the Ministry of Livestock and Fisheries, developed a 5-year national animal health surveillance strategy (2019–2024) (24). The strategy spelt out nine strategic areas of focus to provide a framework for effective and efficient animal health surveillance including active stakeholder engagement and participation. The strategy necessitated the identification of important stakeholders in surveillance and definition and formalization of their roles, responsibilities and expectations.

The first step was a national stakeholder analysis which included stakeholders' responsibilities and likely impact on the surveillance (24). It was a high-level analysis whereby stakeholders were listed in “blanket” categories, e.g., general public, financial institution etc. To understand the details on who and where those stakeholders are and their interaction and influence in animal health surveillance, further analysis at the sub-national level is required, especially at local government authorities. Therefore, this study aimed to explore the existing stakeholder collaborations and influence in animal health at the sub-national level through stakeholder mapping to determine potential leverage points for improving Tanzania's animal health surveillance system. The study specifically focused on (i) identifying stakeholders and their existing relationship with government animal health practitioners, (ii) determining stakeholders' resource and non-resource-based influence on animal health activities (iii) identifying leverage points for collaboration to improve the national animal health surveillance system. It was envisaged that blending national and sub-national level stakeholder analyses can provide insights on stakeholders' collaboration strategies for an effective surveillance system.

## Materials and Methods

### Study Context: Tanzania Animal Health Surveillance System

The study involved the national animal health surveillance system in Tanzania, coordinated by the Ministry of Livestock and Fisheries (MoLF) through the epidemiology unit in the Directorate of Veterinary Services (DVS). The surveillance reporting structure includes eight zonal veterinary centre (ZVCs) and 185 local government authorities (LGAs) in the 139 districts. It is a multi-objective system focused on understanding the disease distribution, the introduction of new strains, risks of disease introduction, and vaccination efficiency. Transboundary animal disease reporting is coordinated by DVS' epidemiology unit, while the One Health coordination desk coordinates zoonotic disease reporting in the Prime minister's office. Surveillance is linked with laboratory service-Tanzania Veterinary Laboratory Agency (TVLA), which falls under the directorate of diagnostic services with 11 zonal laboratories. DVS is also responsible for wildlife surveillance; therefore, if there is any case, it is reported to ZVCs which will communicate to DVS. The primary sources of information for surveillance systems include livestock farmers, zoosanitary border posts and checkpoints, slaughter facilities, livestock markets and veterinary facilities. The livestock field officers are the point of capture for surveillance data.

### Study Areas

**Figure 1:**
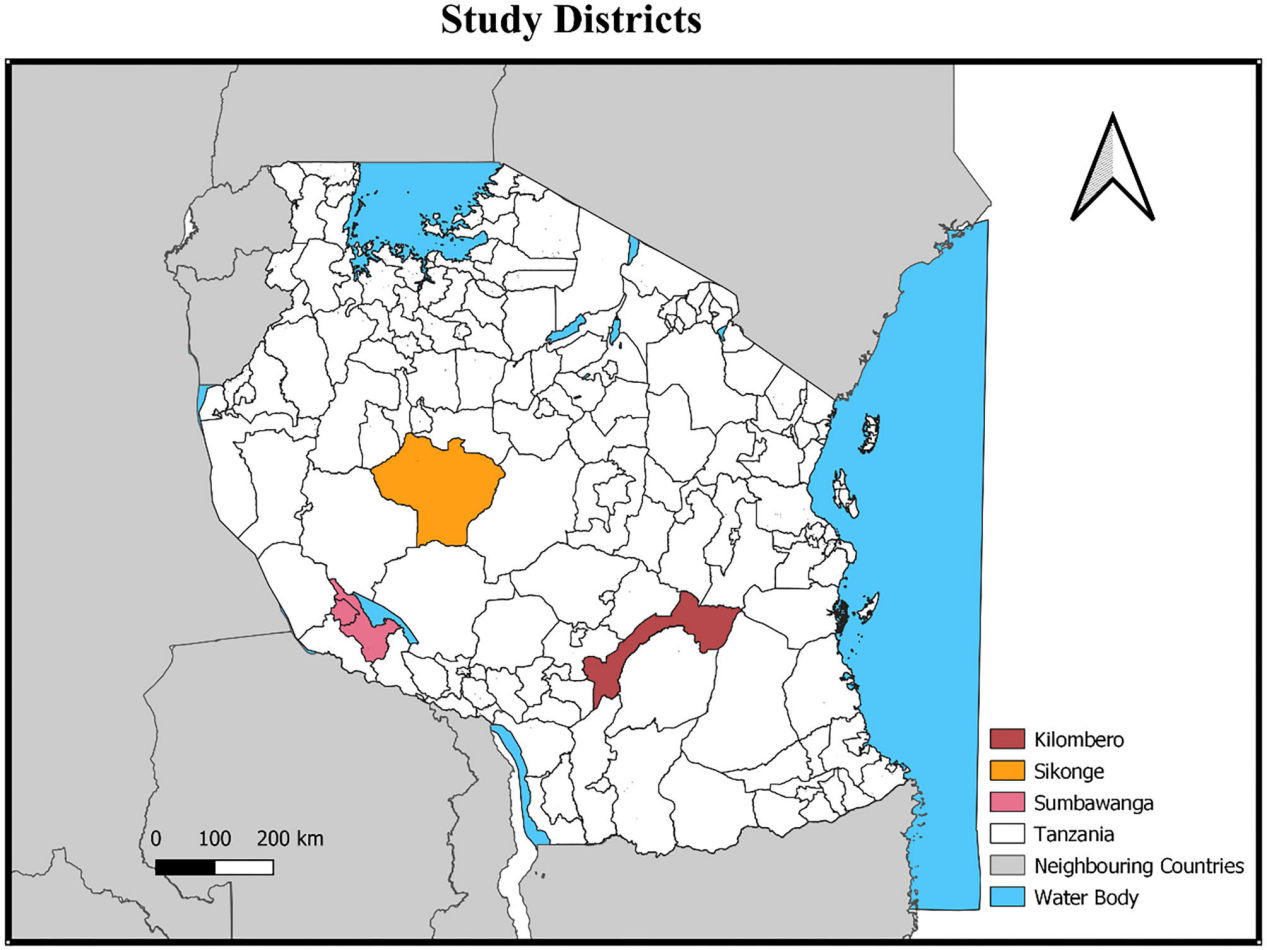
A map showing the study districts (Personal creation using QGIS version 3.12.3-Bucureşti) (25).

The study was conducted in Sumbawanga, Sikonge and Kilombero districts from south-western, western and eastern zonal veterinary centre (ZVCs), respectively (Figure 1). The selection of the ZVCs was purposive and considered practices of reporting disease surveillance data and response to outbreaks. South-western ZVC represented high reporting zones; western ZVC was regarded as medium reporting, while eastern ZVC represented low reporting zones. Then districts were conveniently selected in consultation with officers-in-charge of ZVCs and regional veterinary officers.

### Recruitment of Participants

The study recruited district and field-level staff working in the animal health sector, hereby referred to as Animal health practitioners (AHPs) for a 1-day workshop in each district. The recruited participants included district livestock and fisheries officers (DLFOs), district veterinary officers (DVOs), livestock field officers (LFOs), and ward agricultural extension officers (WAEO) to represent government animal health practitioners. The invited WAEO were only those involved in the delivery of livestock extension services in the areas where there were no LFOs. Participants were selected purposefully, and invited by official letters from the respective local government authorities, followed by telephone calls through DVOs or DLFOs, but it was emphasized that the participation was voluntary. Each study participant gave written consent before the workshop. Thirty-three animal health practitioners from the three districts participated in the study (Sumbawanga= 12, Sikonge= 11, Kilombero = 10).

### Data Collection Methods

The workshop objective was to identify and visualize the list of stakeholders and their interrelationship and influence in animal health in the district that be leveraged to improve the animal health surveillance system. A qualitative study design was adopted in a 1-day workshop in each district using an adapted USAID stakeholder collaboration mapping tool (8). The tool was developed by USAID/Rwanda to graphically depict the USAID's relationship with its key stakeholders. In order to fit the context of the study, the central focus (USAID) was replaced by animal health practitioner. A qualitative study design helps to explore both the process and the outcome of the stakeholder mapping, hence developing a complete picture of the phenomenon as participants reflect on their experiences (26, 27) and gain more insights into the subject (28). The 1-day workshops took place from December 2020 to January 2021.

The tool included a facilitation guide that contained questions to promote discussion during the sessions. The workshops were facilitated by the first author (J.G), supported by two research assistants who were taking notes and organized maps. The USAID stakeholders' collaboration mapping tool involved the following steps: (i) define the objective (ii) identify all stakeholders (iii) take stock of the current relationships (iv) determine resource-based influence (v) determine non-resource based influence and (vi) review and revise the collaboration map. The tool was adapted to reflect the context of this mapping.

***(i) Defining the objective:*** Participants were informed of the objective of the stakeholder mapping. The workshop objective was to identify and visualize the list of stakeholders, their relationship with AHPs and influence in animal health activities in the district that can be leveraged to improve the surveillance system.

***(ii) Stakeholders identification:*** The exercise started by identifying all existing stakeholders working in the animal health sector in a particular district and their areas of operation (village, district, regional, zonal or national). Then, participants grouped identified stakeholders into four categories: government, political leaders, community and private sector and non-government organizations in a quadrant graph.

***(iii) Stock-taking of the current relationships:*** In this step, both the frequency and strength of the interactions were explored. Participants were asked to explain and score the current status of interactions (relationship) between them and each potential stakeholder while creating a collaboration map through discussions and consensus. Participants scored the frequency of their relationship with each identified stakeholder on a 10-point scale as follows: 1–2 = No Interaction, 3–4 = Rare, 5–6 = Intermittent, 7–8 = Regular, and 9–10 = Constant and Consistent. On the collaboration map, the strength of interaction/relations was represented by proximity toward or from the animal health practitioner (which was at the center of the quadrants).

***(iv) Determine resource-based influence:*** The discussion was based on the direct resources such as time, money or staff that stakeholders have invested or can potentially invest in animal health surveillance activities. First of all, participants were asked to point out and discuss all stakeholders with resource-based influence from the list identified in step (ii). After the open discussion, each participant scored each stakeholder on a 10-point scale: 1 = low resource-based influence, 10 = high resource-based influence. The final score was the average from individual participants' scores. On the map, the size of the circle represented the level of resource-based influence of that particular stakeholder.

***(v) Determine non-resource-based influence:*** From the list identified in step (ii), participants were asked to point out all stakeholders with non-resource-based influence in animal health activities. Non-resource-based influence included political power, membership size, access to other resources, leadership in key groups, and name recognition. After an open discussion, each participant scored the stakeholder on a 10-point scale: 1 = low non-resource-based influence, 10 = high non-resource-based influence. The final score was the average from individual participants' scores. On the map, non-resource-based influence was represented by the shade of the circle representing the stakeholder and the darker the circle, the greater the influence.

***(vi) Review and revise the collaboration map:*** After the scores and mapping process, the participants were asked to examine the visual representation of the current relationships and influence and interpret it through open discussions guided by questions such as (a) which stakeholder relationships should be strengthened and why? (b) Which collaborations can be leveraged in strengthening animal health surveillance, and how?

### Data Analysis

Scores were entered into Microsoft™ Excel and analyzed by computing descriptive statistics such as frequency, mean, range and percentages. The scores were also used to create visual maps in the excel sheets embedded in the USAID stakeholder collaboration mapping tool. Additional notes collected during the discussions were summarized and coded manually into Microsoft Word to generate themes.

## Results

### Overview of the Identified Stakeholders in Animal Health Sector at Sub-national Level

Forty-five stakeholders were identified in three districts and grouped into four categories: private sector and non-government organizations (*n* = 16), government (*n* = 16), community (*n* = 9) and political leaders (*n* = 4). Out of the 45 stakeholders identified, only 15 (33%) appeared in all three districts.

***Community stakeholders***: In this category, nine stakeholders were identified. Three stakeholders (religious leaders and reverent elders, livestock farmers' groups and livestock farmers) were mentioned in all three districts. Large livestock commercial farmers were identified in Kilombero and Sikonge, while ward tribunal/community police were identified in Sumbawanga and Sikonge. The following stakeholders were district-specific: Community health committees and community animal health workers (Sumbawanga), dipping committees (Sikonge) and schools (Kilombero).

***Government stakeholders:*** Participants identified 16 stakeholders, of which eight were common in all three districts. Common stakeholders were: Ministry of Livestock and Fisheries (MoLF), district veterinary officers (DVOs), regional secretariat-livestock advisor (RS-LAs), regional commissioners (RCs), district commissioners (DCs), district executive directors/municipal directors (DEDs/MDs), village and ward executive officers (VEOs/WEOs) and health and environmental officers (details of their duties Supplementary Table 1). Other stakeholders identified in more than one district were zonal veterinary centers (ZVCs), Tanzania Veterinary Laboratory Agency (TVLA) (Sikonge and Sumbawanga) and police (Kilombero and Sikonge). District-specific identified stakeholders were Sokoine University of Agriculture (SUA), Tanzania national parks (TANAPA-Udzungwa), national insurance company (NIC) (Kilombero), Mollo-Gerezani and Veterinary Council of Tanzania (VCT) (Sumbawanga).

***Political leaders: Four*** political stakeholders were identified; two appeared in all three districts: village chairpersons and ward councilors. Council chairpersons and members of parliament were mentioned in Kilombero and Sikonge districts.

***Private sector and non-government organizations (NGOs):*** This was the most diverse category, with 16 stakeholders identified. Veterinary shops and animal slaughterers' associations were the most commonly identified stakeholders across the three districts. USAID-*Lishe Endelevu (*sustainable nutrition*)* project and skin and hide processors were identified in Kilombero and Sumbawanga, while livestock business associations were mentioned in Sikonge and Sumbawanga. The majority of the stakeholders (12/16) were district-specific as follows: Ifakara Health Institute (IHI), Caritas and Msabi (Kilombero), *Watu, Simba na Mazingira*/People, Lions and Environment (WASIMA), Ipole-wildlife management area (JUHIWAI- *Jumuiya ya Uhifadhi wa Wanyamapori Ipole*) and Promoting Sustainable Practices to Eradicate Child Labor in Tobacco (PROSPER) project (Sikonge) and Sumbawanga Agricultural and Animal Food Industries Ltd (SAAFI), Milk processing industry-OTC, Efatha, Asas and Monrovian church (Sumbawanga).

### Relationship Strength Between Stakeholders and Animal Health Practitioners

**Figure 2:**
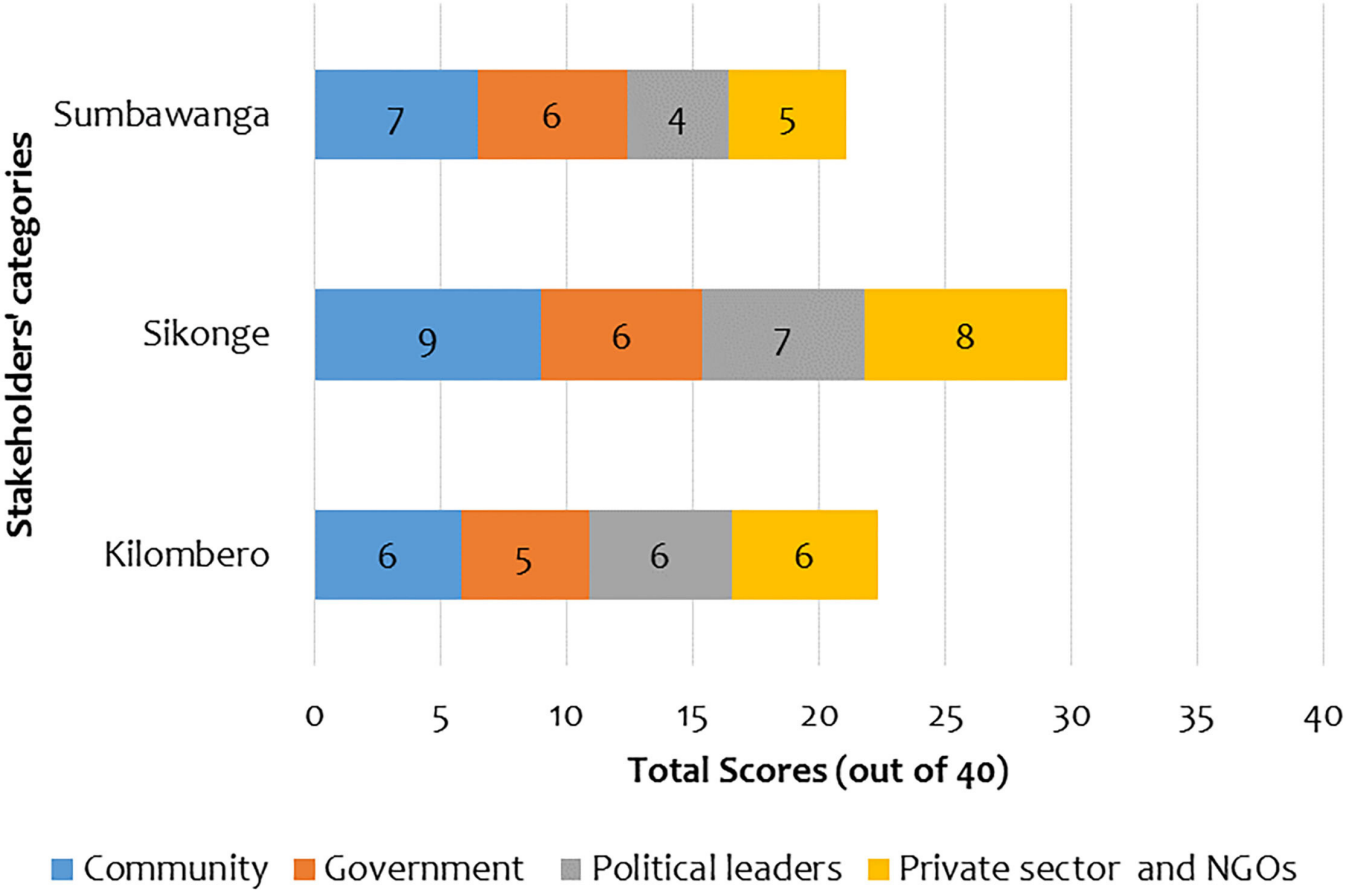
Relationship scores by district scored by the Animal Health Practitioners (AHPs).

Generally, there was a stronger relationship between AHPs and community stakeholders and the private sector and NGOs than in other categories. The average relationship strength scores between AHPs and community stakeholders was (6.8/10, range = 3–10), private sector and NGOs (6.0/10, range = 2–9), government (5.1/10, range = 1–10), and political leaders (5.0/10, range = 1–9). Figure 2 gives the breakdown of stakeholder relationship as scored by AHPs in each district. Sikonge had the strongest relationship with all stakeholders, followed by Kilombero. Sumbawanga was relatively stronger in communities and government categories compared to Kilombero.

### Stakeholders With Resource-Based Influence on Animal Health Activities

**Figure 3:**
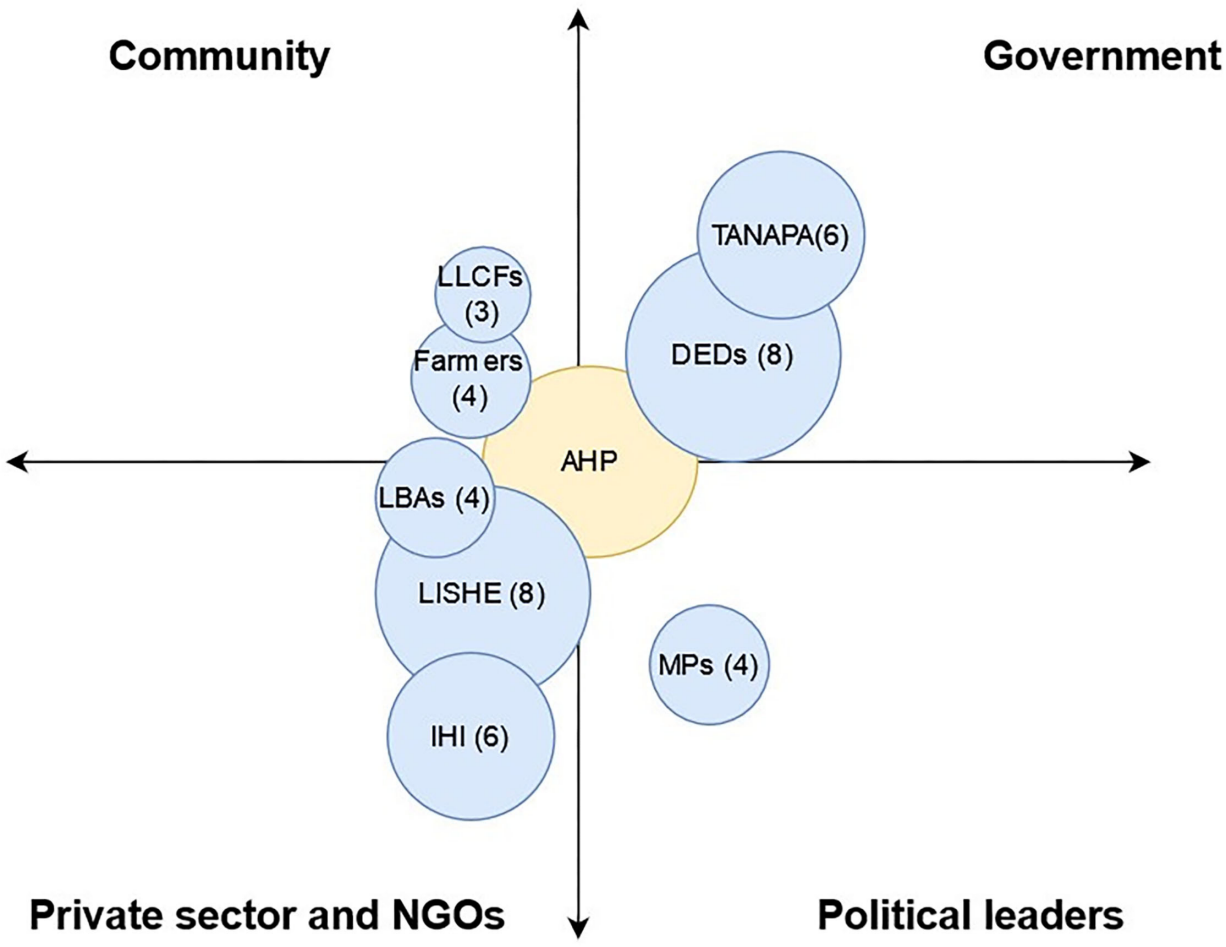
Stakeholders with resource-based influence on animal health activities as scored by Animal Health Practitioners (AHPs). The size of the circle represents the level of resource-based influence of that particular stakeholder corresponding to the scores in brackets. The proximity between AHP circle and others represents the relationship strength. (LLCFs, large livestock commercial farmers; DEDs, district executive directors; TANAPA, Tanzania national parks; LBAs, livestock business associations; LISHE, Lishe endelevu; IHI, Ifakara health institute; MP, Member of Parliament).

Out of 45 stakeholders identified across all the districts in four categories, only eight of them were reported to have resource-based influence, which in most cases was through financial support (Figure 3). Stakeholders were district-specific except DEDs, who appeared in all three districts. In the community category, both small and large livestock farmers were mentioned in Sikonge. The *Lishe Endelevu, a* USAID sponsored nutrition project was found to have a high resource-based influence in providing financial and technical support in Sumbawanga and Kilombero. Apart from members of parliament who have designated constituency development catalyst funds, political leaders had no resource-based influence in animal health activities.

### Stakeholders With Non-resource-based Influence on Animal Health Activities

**Figure 4:**
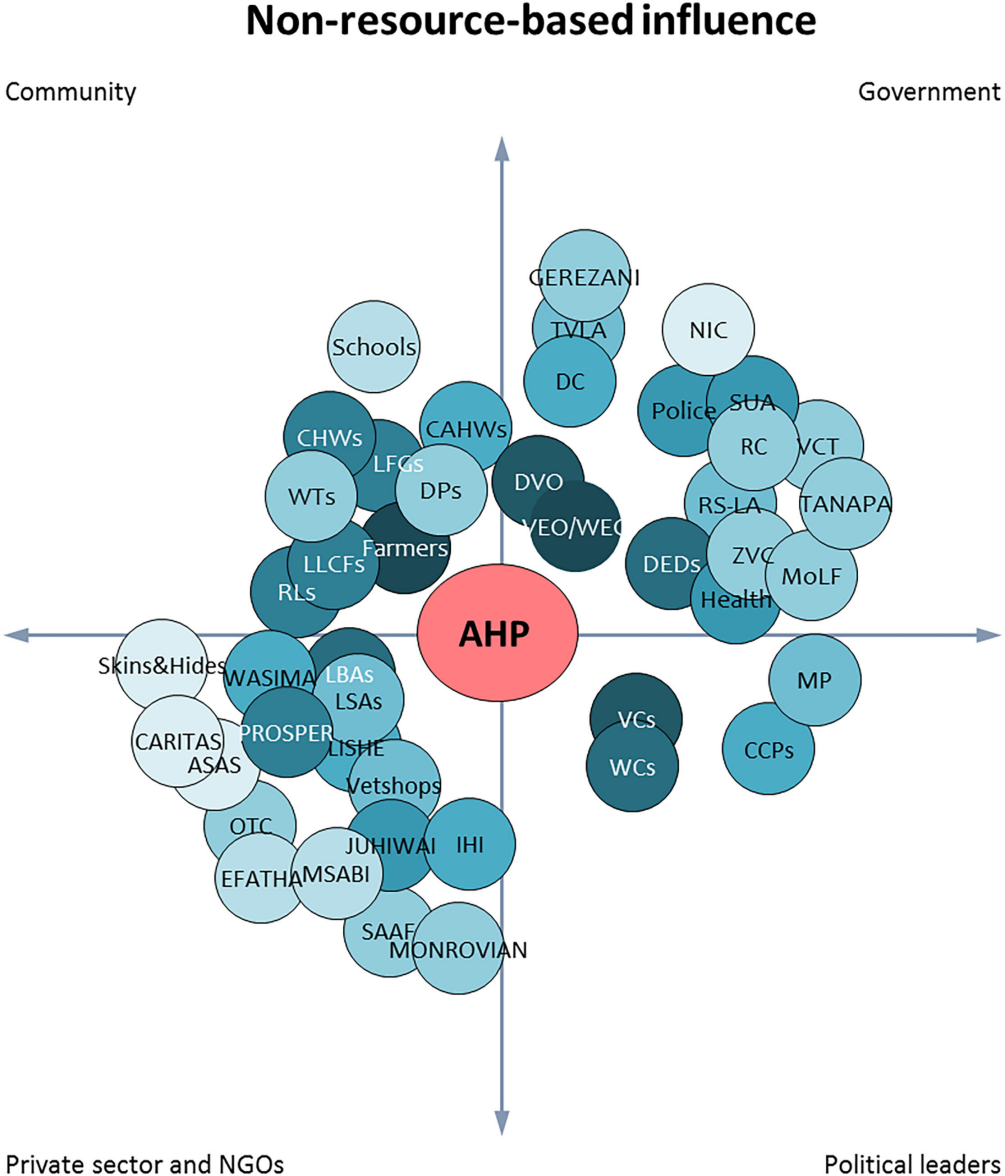
Stakeholders with non-resource-based influence on animal health activities as scored by Animal Health Practitioners (AHPs). The shade of the circle represents the level of non-resource-based influence of that particular stakeholder. The darker the shade the higher the influence. The proximity between AHP circle and others represents the relationship strength. (RL, Religious leaders; LFG, Livestock farmers' groups; LLCFs, Large livestock farmers; CHWs, Community health workers; CAHWs, Community animal health workers; DPs, Dipping committees; WTs, Ward tribunals; DVO, District veterinary officer; RS-LA, Regional secretariat-Livestock advisor; DEDs, District executive director; VEO, Village executive officer; WEO, Ward executive officer; Health, Health officers; ZVC, Zonal veterinary centre; TVLA, Tanzania Veterinary Laboratory Agency; VCT, Veterinary council of Tanzania; MoLF, Ministry of livestock and fisheries; SUA, Sokoine University of Agriculture; TANAPA, Tanzania National Park; NIC, National Insurance Cooperation; DC, District commissioner; RC, Regional commissioner; VCs, Village chairpersons; WCs, Ward councilors; CCPs, Council chairpersons; MP, Member of parliament; LISHE-Lishe Endelevu; SAAFI, Sumbawanga Agricultural and Animal Food Industries Ltd; LBA, Livestock business associations; LSAs, Livestock slaughter associations; WASIMA, Watu, Simba na Mazingira/People, Lions and Environment; PROSPER, Promoting Sustainable Practices to Eradicate Child Labor in Tobacco; Skin and hides, Skin and hide processors; JUHIWAI, Jumuiya ya Uhifadhi wa Wanyamapori Ipole; IHI-Ifakara Health Institute; OTC, OTC-Milk processing industry).

The average scores indicate that political stakeholders and government had relatively higher non-resource-based influence than the community and private sector stakeholders (political leaders = 6.5/10, government = 4.8/10, community = 3.9/10, and private sector = 3.8/10). The most influential stakeholders were livestock farmers, VEOs and WEOs, village chairpersons, livestock business associations (cut off point is 5). Figure 4 depicts the majority of the government stakeholders have greater influence (darker circles) compared to other categories. The private sector is a highly diverse category with more stakeholders, but only 6 of them scored 5 points and above in non-resource based influence.

### Stakeholders' Relationship Strength vs. Influence on Animal Health Activities

**Figure 5:**
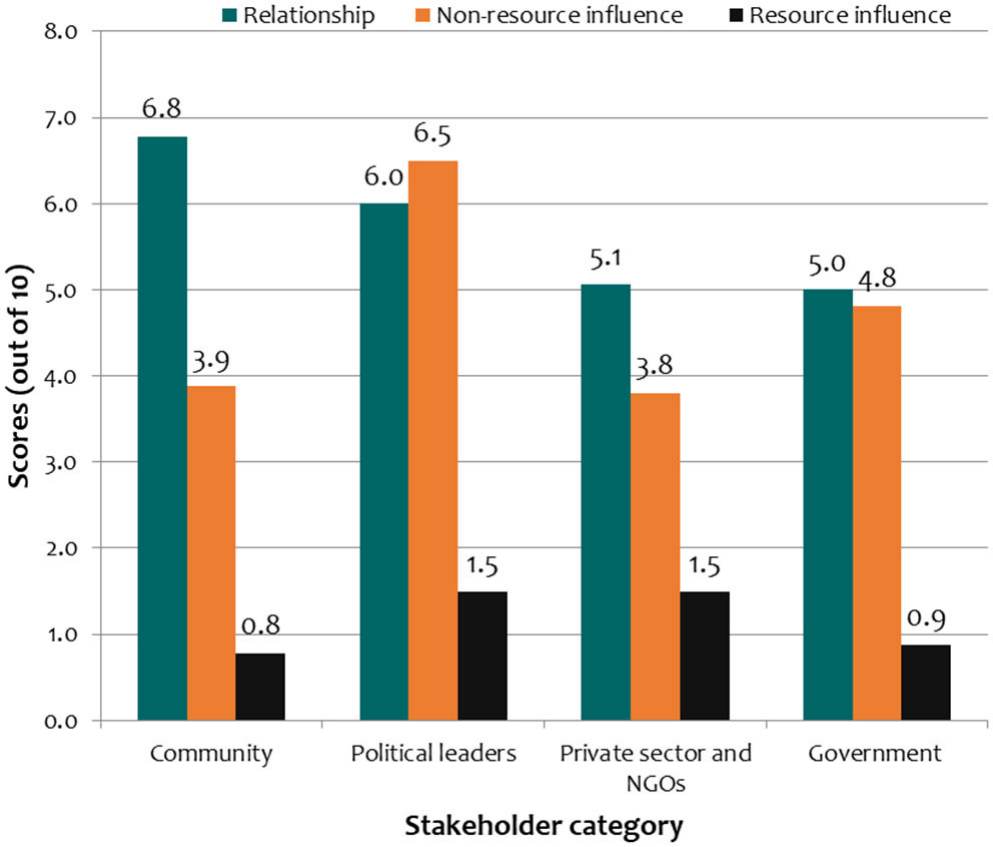
Comparison of scores on relationship strength, resource-based and non-resource-based influence of community stakeholders in animal health activities.

The comparison between relationship strength and influence shows that the relationship strength scores were generally higher than scores on the influence on the same scale of measurement (1 = lowest, 10 = highest). The resource-based influence scores were the lowest in all the four categories of stakeholders. Despite having the strongest relationship with AHPs, community stakeholders were scored the lowest in the resource-based influence on animal health activities. Political leaders were scored higher in non-resource-based influence despite the relatively lower interaction with AHPs. On the other hand, government stakeholders were scored almost equally in the relationship and non-resource-based influence (Figure 5).

### Role of the Stakeholders in Animal Health Sector and Their Potential Contribution to Surveillance

It was found that the role of stakeholders varied distinctively across the categories. Community-level stakeholders were primarily involved in community mobilization and sensitization on animal health activities such as mass vaccination, dipping, and educational campaigns. Ward tribunals or community police have been used in conflict resolutions and law enforcement. Interaction between government stakeholders and AHPs varied greatly from one to another. DVOs and RS-LAs provided supervision and technical support to the field level officers while acting as the linkage between local government authorities and ministries. DEDs/MDs were the facilitators of sectoral activities because they are heads of the staff, in charge of day-to-day activities, and custodians of the budget of the local government authorities. VEOs/WEOs were more into the supervision of operations, community mobilization, revenue collection and exercise executive power in their jurisdiction areas. In case of disease investigation, AHPs through DVOs collaborated with TVLA and ZVC and institutions such as SUA. MoLF and VCT operated at a high level, whereby the former provides policy and guidelines on animal health and surveillance while the latter oversees animal health professionals' performance. AHPs worked closely with health and environmental officers, especially in meat inspection and issues related to public health. Police were consulted for law enforcement, especially during livestock markets and illegal slaughtering, while DC and RC had an extra role in maintaining laws and orders, including regulating livestock movements. TANAPA-Udzungwa provided support in livestock vaccination while NIC advocated for livestock insurance.

Regardless of their levels, political leaders were viewed as catalysts because their words have a substantial impact; they could positively or negatively influence people driven by their interest in the matter and engagement level. For instance, village chairpersons and ward councilors had been primarily engaged in mobilization and decision-making while members of parliament also took part in fundraising activities. On the other hand, private sector and NGO stakeholders had been collaborating with AHPs to provide veterinary and animal production services, including vaccination, supply of breeding animals, disease control and public health management.

Table 1 shows the summary of the roles played by the stakeholders identified at the sub-national level and their potential contribution to animal health surveillance. However, it was also pointed out that for their contribution to be of value, some issues needed to be considered, including integration of veterinary services for both private and government service providers, a direct link between line ministry (MoLF) and AHPs at the field level, the political will of the politicians to positively influence changes and prioritization of animal health sector in the local government authorities.

**Table 1:** Summary of stakeholders' roles in animal health sector and their potential contribution to surveillance.

**Stakeholder category**	**Role in animal health sector**	**Contribution to animal health surveillance**
Community	- Community mobilization and sensitization e.g., LFGs, CAHWs - Conflict resolution and law enforcement e.g., ward tribunals and community police - Educational campaigns	- Fostering the flow of surveillance data from the community - Accurate and timely reports
Government	- Supervision and technical support to AHPs e.g., ZVCs - Facilitation of sectoral activities e.g., DEDs - Disease investigation e.g., TVLA and SUA - Policy and guidelines- MoLF - Law enforcement- Police - Maintain law and order e.g., DCs - Vaccination e.g., TANAPA	- Support technical aspects of surveillance - Financing surveillance activities - Prompt testing and proper diagnostic tests - Adherence to surveillance protocols - Timely and accurate reports - Disease control through regulation of livestock movements - Disease control by vaccination
Political leaders	- Mobilization and decision making e.g., Councils' chairpersons - Catalysts and resources mobilization e.g., MPs	- Financing surveillance activities - Timely and accurate reports
Private sector and NGOs	- Provision of veterinary services - Vaccination - Public health management - Resource-based support	- Timely and reliable reports - Prompt and accurate tests and diagnostic services - Disease control through vaccination - Financing surveillance activities

## Discussion

This study aimed at exploring the existing stakeholders' collaborations and influence in animal health at the subnational level through stakeholder mapping to determine potential leverage points for improving the national animal health surveillance system. Participants identified and categorized 45 stakeholders involved in animal health activities, the majority of them being government stakeholders and private sector and NGOs. Among the four categories, community stakeholders had the strongest relationship with animal health practitioners. The private sector had a relatively higher number of resource-based influential stakeholders, while political leaders have more non-resource-based influence. The list of identified stakeholders was diverse across the districts as the common mentions were only 33% of the total; private sector and NGOs being the most diverse group with only 2/16 common stakeholders. This may be attributed to the nature of the governance, geographical location of the stakeholders, collaborative culture and political influence (29). Stakeholders were found to play diverse roles in animal health activities while contributing or have the potential to contribute to animal health surveillance. To the authors' knowledge, this is the first sub-national level stakeholder mapping for the animal health surveillance system in Tanzania to examine stakeholders' collaboration with government field staff and their influence. Therefore, the findings of this study complement the national-level stakeholder analysis, which can inform stakeholders' collaboration strategies for an effective surveillance system.

We chose the least conventional stakeholder mapping approach by focusing on animal health practitioners who are regarded as the frontline workers. The approach enabled to get practitioners' perspective on the stakeholders they were interacting with or who may positively or negatively influence the implementation of animal health activities. However, the tool didn't look on the power structures which would have been very important in assessing the ways stakeholders engage with each other, and what need to be changed for effective collaboration. Due to resource constrains, the mapping was only limited to the existing collaboration and influence and how could be leveraged to improve animal health surveillance. Future stakeholder mapping could benefit from combined analyses especially on assessing the impact of such collaboration in the performance of the animal health surveillance system.

Acceptability is one of the attributes of the animal health surveillance system (30), and the strong relationship between community stakeholders and animal health practitioners can be leveraged to achieve that. Several studies support community animal health workers in accelerating disease reporting and the need to institutionalize them (31–33). At the same time, Padmawati et al. (34) highlighted the bridging roles of community and religious leaders in health interventions such as vaccination. Tanzania's animal health surveillance is primarily passive and heavily relies on the reports from livestock field officers and farmers who seek veterinary services; therefore, the system's performance depends on their awareness and capability to detect disease or unusual syndromes and incentive to report. Participants pointed out how non-resource influence of stakeholders, such as farmers' groups and committees, has helped in mobilization and sensitization of matters related to animal health. Farmers play a central role in biosecurity and disease management, but their involvement has always been low (21). For the community-level stakeholders to cooperate in surveillance activities, they have to feel that their needs are being catered, hence there is important to make a compromise between national and local interests (2). The system may benefit from smooth community mobilization and sensitization, locally mobilized resources and expanding the horizon of surveillance data sources by tapping on the existing influences and interactions between stakeholders and practitioners.

Political leaders were described to be powerful in mobilization and sensitization due to political capital despite their low resource-based influence. That attribute can positively or negatively affect the implementation of animal health activities, especially in disease control interventions. The influence of stakeholders does not necessarily mean they will use it to promote changes (35). For instance, there have been reported incidences of political interference in Tanzania during the implementation of activities or law enforcement (36, 37). Therefore, it is essential to understand factors that may influence stakeholder participation, including perceived benefits and costs. It was also interesting to find that the private sector and NGOs had more resource-based influence than other stakeholders, including the government. Since the mid-eighties, the government of Tanzania has left most animal health services, including control of non-trans-boundary animal diseases and supply of veterinary medicine and other inputs, to the private sector and retained regulatory and public support functions (38–40). Weak collaboration between public and private sector actors had been reported to hinder the provision of animal health services (41). However, this study has contrasting findings given the high number of private and non-government stakeholders listed, their relationship with animal health practitioners and resources influence on animal health activities. To foster further collaboration with the private sector and NGOs in animal health services and primarily surveillance, the following can be done: joint public-private initiatives, e.g., surveillance programmes, regular discussions on the areas of collaboration for sustainable partnership, leveraging national and international public-private partnership guidelines to establish collaboration strategies and proper acknowledgment of private actors' contribution to animal health services including surveillance.

The results confirmed the distinct roles of local and central governments in animal health services and surveillance in general. Animal health practitioners reported interacting more with DEDs and VEOs/WEOs than other stakeholders in the same category, including the MoLF, the custodian of animal health surveillance. According to the animal health surveillance strategy (2020–2024) local government authorities (LGAs) are responsible for recruiting animal health service providers, fund mobilization, data collection and disease reporting, technical backstopping and enactment of by-laws disease surveillance at the district level (24). On the other hand, MoLF is responsible for coordination, high-level technical support, capacity building and data collection, analysis and dissemination of animal health-related information (24). However, this institutional arrangement may be flawed because the performance of surveillance activities in the district is upon the discretion and interest of that particular council management team to allocate adequate budget and field staff who are on the frontline. Meanwhile, there is no direct link between MoLF as the sectoral ministry and local government authorities except through President's office-Regional and local government authorities, which plays a coordination role (42). This institutional structure may compromise subnational-level coordination, and sectoral accountability hence affects the implementation of surveillance activities.

Limited cross-sectoral collaboration was spotted during the mapping, whereby only three stakeholders were identified and scored. A modest interaction with health and environmental officers was reported while IHI and *Lishe Endelevu* project provided significant resource-based support in zoonotic diseases and nutrition, respectively. In the light of One Health, this is a step toward desired multi-sectoral collaboration, which is recognized as the key ingredient to solving complex health problems. There have been concerns about the limited engagement of the livestock sector in One Health activities, yet it is the epicenter of emerging zoonotic pathogen threats and food contamination (33, 43). An animal health surveillance system can benefit from such collaboration through resource and data sharing and joint surveillance activities hence improve early disease detection and outbreak prevention.

Animal health surveillance system may benefit from the existing collaborations and influence by leveraging the diverse roles of the stakeholders and their non-resource-based influence on animal health activities. They can contribute to surveillance by providing accurate and timely data, disease control through vaccination and educational campaigns, financing surveillance activities and support diagnostic services and tests. In light of the study findings, here are some of the proposed leverage points for improving animal health surveillance and response in Tanzania: (i) integration of surveillance into animal health services instead of treating as a separate activity, (ii) harnessing non-resource influence through constant engagement with stakeholders especially on participatory epidemiology, (iii)government should take leading role in coordinating stakeholders' efforts and create bridges between local and central authorities for shared responsibilities and accountability in surveillance and disease control (iv) paying attention to both procedures and interpersonal relationship among stakeholders as key factors for collaboration as demonstrated across the districts (v) Clear operational processes in order to reduce conflicts and power struggles during implementation of activities and (vi) Incentivize collaboration to encourage right behavior toward animal health surveillance such as prompt feedback to reporters and acknowledgment of stakeholders who contribute their efforts to particular course. It is also important to create inclusive multi-stakeholders platforms with a balanced share of resource and non-resource-based influential stakeholders for dialogues, information sharing, planning and collective interventions.

It should be noted that the study focused on three districts which were purposively selected for exploration. Therefore, it will be difficult to use these findings to make general inference for the entire country. However, the findings may guide identification and analysis of stakeholders in similar context and also inform future studies on stakeholder mapping. Since the mapping was based on practitioner's perspective, the information from participants might have some elements of subjectivity as a result of their experience and interactions with stakeholders. Stakeholder analysis on its own may not lead to system change due to many other factors. Therefore, it is important to use systems thinking to unpack those causal drivers for the operational system.

## Conclusion

This article presents the importance of subnational-level stakeholders mapping and how animal health surveillance can leverage their collaboration and influence to improve the system's early disease detection and response efficiency. The findings of this study complement the national-level stakeholder analysis, which can inform stakeholder collaboration strategies for an effective surveillance system. The diversity in the identified stakeholders across the districts suggest that collaboration are contextual and socially constructed. Despite many identified stakeholders, very few of them had resource-based influence compared to non-resource-based influence, but they played diverse roles in animal health activities which can also contribute to surveillance. The next step would be to analyse interests, power dynamics and priorities of the identified groups with respect to animal health surveillance in order to benefit from their presence and interactions at sub-national level.

Stakeholder mapping is a step toward effective stakeholder engagement but may not automatically lead to the improvement of animal health surveillance. Other aspects that should be considered include integration of surveillance into animal health services, clear operational processes, constant engagement, coordination and incentivization of stakeholders, to mention a few. The position of local government authorities on animal health surveillance is indisputable; therefore, to harness their influence, a coordination mechanism that aligns national surveillance objectives with local priorities is indispensable. The study also demonstrated a new perspective on collaborative stakeholder mapping, especially at sub-national levels, involving government field staff. Through this kind of analysis, national animal health surveillance may benefit from resource and non-resource influence and stakeholder interactions.

## Data Availability Statement

The raw data supporting the conclusions of this article will be made available by the authors, without undue reservation.

## Author Contributions

JG conceptualized the study with critical inputs from BH, CS, EK, JM, MR, and SK. JG collected, analyzed, and interpreted data and wrote the first draft of the manuscript. BH, CS, EK, JM, MR, and SK provided oversight throughout data collection, analysis and interpretation of the results. BH, CS, EK, JM, MR, and SK substantively revised the first draft of the manuscript and subsequent modifications. All authors contributed to the article and approved the submitted version.

## Funding

The study is part of research on the development of a prototype for cost-effective integration of animal health surveillance systems in Tanzania supported by the Government of Tanzania and World Bank, grant no. PAD 1436 through SACIDS Africa Centre of Excellence for Infectious Diseases of Humans and Animals in Southern and Eastern Africa (SACIDS-ACE). The funder had no role in the design of the study, data collection, analysis and interpretation or preparation of this manuscript.

## Conflict of Interest

The authors declare that the research was conducted in the absence of any commercial or financial relationships that could be construed as a potential conflict of interest.

## Publisher's Note

All claims expressed in this article are solely those of the authors and do not necessarily represent those of their affiliated organizations, or those of the publisher, the editors and the reviewers. Any product that may be evaluated in this article, or claim that may be made by its manufacturer, is not guaranteed or endorsed by the publisher.
