# Supplementary material for: Leveraging Sub-national Collaboration and Influence for Improving Animal Health Surveillance and Response: A Stakeholder Mapping in Tanzania
Source: Front Vet Sci. 2021 Dec 13;8:738888. doi: 10.3389/fvets.2021.738888 (PMC8710487; doi:10.3389/fvets.2021.738888)
Supplement: Supplementary file 1 [file Table_1.DOCX]

Supplementary table 1. Duties and responsibilities of various government stakeholders

| Name of the government stakeholder | Duties and responsibilities |
| --- | --- |
| District veterinary officer (DVO) | Veterinarian in-charge in a district. Is responsible for overseeing animal disease control issues and report to Director of Veterinary Services |
| Regional secretariat- Livestock Advisor (RS-LA) | To provide expert facilitation on Livestock sector to the Local government authorities (LGAs) in the region |
| Regional commissioner (RC) | Principal representative of the government in the region responsible for securing the maintenance of law and order and determining the specific direction of efforts in implementing the general policies of the government in the region  Assist local government authorities in the  region to undertake and discharge their responsibilities by providing and securing enabling environment for the successful performance of their duties |
| District commissioners (DC) | Responsible for securing the maintenance of law and order in the district and determining the specific direction of efforts in implementing the general policies of the government in the district  Assist local government authorities in the  region to undertake and discharge their responsibilities by providing and securing enabling environment for the successful performance of their duties |
| District Executive Director (DED)/Municipal Director (MD) | Is the chief executive and administrative officer of the district or municipal council  Responsible for coordinating all activities in the council and its committees |
| Village Executive Officer (VEO)/ward executive officer (WEO) | Responsible for the day-to-day management of the village/ward development issues and conflict resolution |
